# Study on Multi-Factor Coupling Fatigue Properties of Weathering Steel Welded Specimens

**DOI:** 10.3390/ma18194551

**Published:** 2025-09-30

**Authors:** Shuailong Song, Guangchong Qin, Tao Lan, Zexu Li, Guangjie Xing, Yanchen Liu

**Affiliations:** 1 School of Civil Engineering, Qingdao University of Technology, 777 Jialingjiang East Road, Huangdao District, Qingdao 266520, China; ovossl@163.com (S.S.); yanzhao3255@163.com (Y.L.); 2 CSSC International Engineering Co., Ltd., 1 North Courtyard, Shuangqiao Middle Road, Chaoyang District, Beijing 100121, China; qinguangchong@126.com (G.Q.); lizexu@csic602.com.cn (Z.L.); 3 School of Civil Engineering, Xian University of Architecture & Technology, No. 13 Yanta Road, Beilin District, Xi’an 710055, China; xingguangjie@cmhk.com

**Keywords:** Q500qENH weathering steel, high-altitude low-temperature corrosion, fatigue test, crack propagation, fatigue life degradation mechanisms, scanning electron microscopy

## Abstract

Environmental factors significantly affect the fatigue performance of weathering steel welded components in high-altitude, low-temperature corrosive environments. This study conducted multi-factor-coupled constant-amplitude fatigue tests on Q500qENH weathering steel V-groove welded joints and built an equivalent finite element model using test data to explore key influencing factors under multi-condition coupling. Results show that stress level most significantly affects fatigue performance, followed by corrosion duration, then ambient temperature, with influences decreasing in turn. Analyzing 18-day cyclic immersion corrosion morphology predicts 21-year outdoor corrosion in plateau regions, providing a reliable method for long-term exposure prediction. Finite element simulations confirm that low temperatures improve slightly corroded specimens’ fatigue performance by 20%, but damage accumulates before optimal service. This study offers key parameters for safe design of high-altitude weathering steel welded components.

## 1. Introduction

The western plateau region of China is characterized by pronounced annual and diurnal temperature variations [1], abundant summer rainfall, and intense solar radiation, all of which adversely affect the service life of steel bridges in high-altitude areas [2,3]. Compared with traditional coated steel, weathering steel incorporates corrosion-resistant alloying elements (e.g., Cu, P, Mo, Cr, Ni) [4], whose corrosion products and morphological characteristics depend significantly on alloy composition and atmospheric conditions [5]. In corrosive environments, this weathering steel forms a double-layered rust structure [6]: the outer layer is a porous iron oxide layer (with compounds like FeO and Fe_2_O_3_), while the inner layer is a dense amorphous FeOOH protective film. This process facilitates forming a protective passive film on the steel surface, which effectively prevents the underlying alloy from further corrosion [7]. This film effectively isolates the uncorroded steel substrate from further contact with corrosive media, significantly reducing ongoing corrosion rates [8,9]. Thus, over a 100-year service life, the adoption of uncoated weathering steel can reduce coating costs by more than 6.00%, rendering it an increasingly preferred material in bridge construction [10].

Corrosion reduces the cross-sectional area of weathering steel, and induces stress concentrations at pits, thereby degrading fatigue performance. For instance, Zhang et al. [11] conducted fatigue tests on corroded Q345NH weathering steel, finding 22.60% to 38.30% degradation in fatigue strength compared to uncorroded specimens. When environmental temperatures fall below the material’s ductile-to-brittle transition threshold, further cooling reduces steel plasticity, diminishing alloy ductility and shortening fatigue life. Notably, Zhang et al. [12] performed high-cycle fatigue tests on Q500qENH weathering steel across temperatures from 19.85 °C to −60.15 °C. Their research revealed that within this range, decreasing temperatures cause metal atoms to enter lower-energy states, increasing dislocation migration resistance and cold brittleness. This enhances resistance to plastic deformation while paradoxically extending fatigue life. Within a certain range, an increase in the metal’s resistance to plastic deformation can extend its fatigue life; if the resistance to plastic deformation becomes excessively strong, it may lead to increased brittleness, thereby shortening the metal’s fatigue life. The extension of fatigue life typically requires the metal to release stress concentration through localized plastic deformation under cyclic loading. Excessive resistance to plastic deformation may inhibit this stress release, instead accelerating crack initiation.

In the application scenarios of weathering steel materials, welding of weathering steel components is frequently required. Although the introduction of corrosion-resistant alloying elements can enhance the corrosion resistance of weathering steel, some of these elements are affected by high welding temperatures, causing the microstructure to gradually deteriorate the weldability of weathering steel. During the welding process, this leads to segregation of alloying elements and gradient differences in cooling rates [13], resulting in defects such as lack of fusion and porosity in weathering steel components. The surface of the weld seam exhibits a highly irregular state, with each irregularity forming a notch. Each notch has both a local and global effect, as the number of notches influences the number of cracks triggered. Consequently, this causes a reduction in the fatigue life of the welded joint specimens [14]. After welding, the cooling process inevitably generates welding residual stresses in the weld zone. These stresses may induce stress concentration effects between the weld zone and the base material zone, further promoting crack initiation and propagation. Therefore, when monotonic stress is applied to welded joint specimens in fatigue testing, the location of maximum stress is prone to occur in the transition zone between the weld seam and the base material [15]. Zhang [16] conducted a comparative study on the fatigue performance of Q345NH weathering steel base metal, butt joints, and cruciform joints. The results showed that welding materials and welding process parameters exert a significant influence on the fatigue performance of weathering steel. The toughness degradation of weld metal was identified as the primary factor leading to reduced fatigue life of joints. Liao et al. [17] conducted fatigue crack growth tests on Q345qD bridge steel and its butt welds across multiple temperature zones (room temperature, −20.15 °C, and −60.15 °C). The results demonstrated that while decreasing environmental temperature reduced the crack growth rate in base metal, the weld joint region exhibited a significant increase in crack growth rate with temperature reduction due to the coupled effects of microstructural inhomogeneity and residual stresses. This reveals the unique evolution mechanism of fatigue damage in welded joints under low-temperature environments.

In summary, this study investigates crack propagation in V-groove welded joints of Q500qENH weathering steel under plateau low-temperature corrosive environments using an orthogonal experimental design including temperature, corrosion duration, and stress level. Constant-amplitude fatigue tests were conducted on V-groove welded joints specimens combined with SEM fractographic analysis to elucidate the damage evolution mechanisms governing crack initiation, propagation paths, and fracture characteristics. An initial-defect-containing finite element model was established based on experimental data to simulate crack propagation processes and validate life prediction accuracy. This work provides theoretical support for safety assessment of welded weathering steel structures in plateau regions, offering significant implications for enhancing structural reliability under extreme environmental conditions.

## 2. Experimental Design

To investigate the variation patterns of fatigue life in weathered steel specimens with V-groove welded joints affected by corrosion under high-altitude low-temperature environments, fatigue tests were conducted on corroded V-groove welded joint specimens of weathered steel based on orthogonal experimental design. The fatigue test results were calculated and summarized, followed by comparative analysis to identify the optimal combination of influencing factors for fatigue life.

### 2.1. Specimen Dimension Design and Parameters

**Figure 1:**
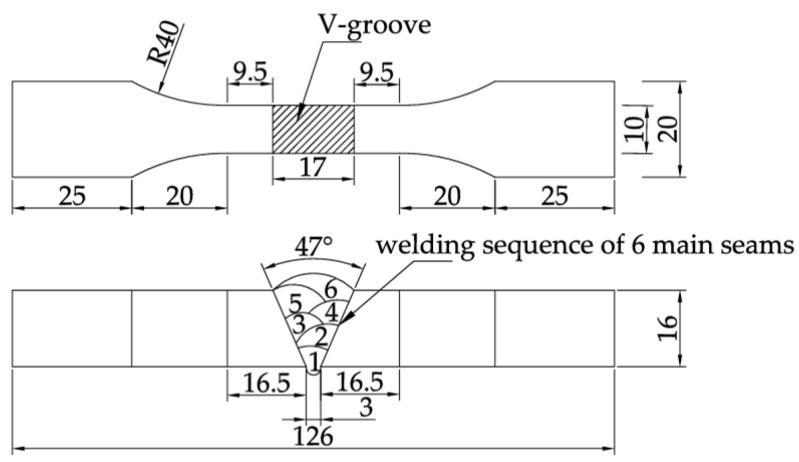
Weathering steel specimen with V-groove welded joint.

This test utilized 16 mm thick Q500qENH weathering steel plates to prepare V-groove welded joint specimens, with geometric dimensions shown in Figure 1 and base metal chemical composition detailed in Table 1. To address the welding characteristics of this high-strength bridge steel, JWER60NHQ solid wire (GMAW) with a diameter of 1.20 mm and JWS60NHQ submerged arc welding wire (SAW) with a diameter of 4 mm were selected. The chemical compositions of both wires are specified in Table 2. Take the chemical compositions of the weathering steel base metal and weld metal (Table 1 and Table 2) from the actual production data provided by the manufacturer. Before factory delivery of the specimens, follow the weld inspection methods for welded specimens specified in GB/T 11345-2023 [18], and conduct UF Union-PXUT-350BPLUS (Nantong City, Jiangsu Province, China) ultrasonic flaw detection on the welds of all weathering steel V-groove welded joint specimens in each group to avoid defects such as insufficient weld fusion. A hybrid welding method combining gas metal arc welding (GMAW) and submerged arc welding (SAW) was employed. The procedure strictly adhered to the technical requirements of: GB/T 985.1-2008 [19], and GB 50661-2011 [20]. The specific welding parameters of Q500qENH weathering steel V-groove welded joint specimens are presented in Table 3, including welding passes, welding current, welding voltage, and welding speed. Representative welded specimens were visually inspected, and the average weld toe transition radius of the specimen welds was found to be approximately 15 mm, with the weld toe reinforcement around 1.2 mm—both meet the geometric dimension requirements for welded specimen welds specified in GB 50661-2011 [20] (see Table 4). To ensure uniform exposure of the test specimens to the corrosive medium, a surface pretreatment was conducted prior to periodic immersion corrosion testing, following the national standard GB/T 16545-2015 [21]. The pretreatment involved a combination of grinding with an Dongcheng-DCSM02-100E (Nantong City, Jiangsu Province, China) angle grinder and immersion in an acetone solution to remove surface impurities and the dense oxide layer on the metal. After completing the above pretreatment, the standardized specimens were thoroughly dried, followed by dimensional measurement and systematic numbering (see Table 4 for details).

**Figure 2:**
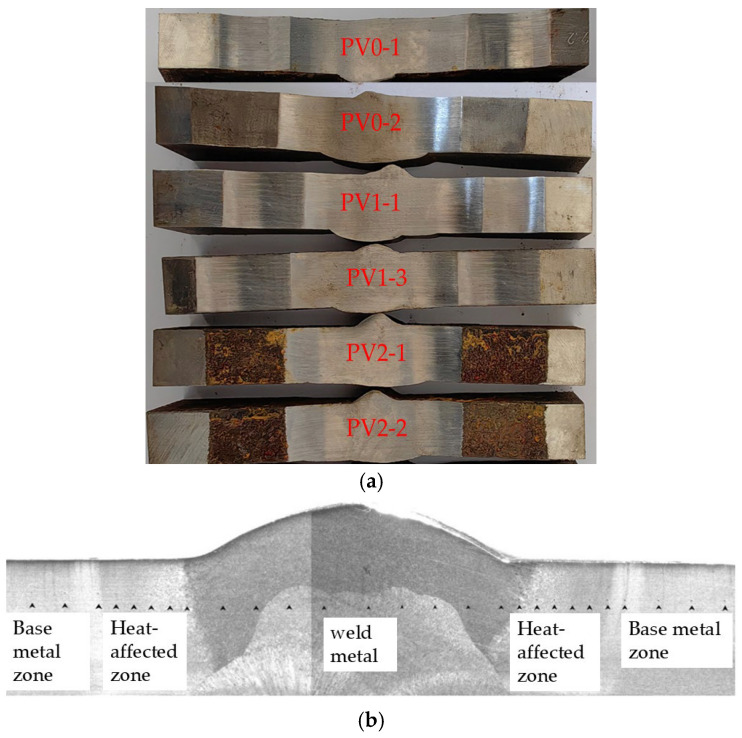
(**a**) V-groove welded joint specimens of weathering steel. (**b**) Morphology of V-groove.

The specimen numbering adopts a four-level coding system: “test type-specimen form-corrosion cycle-parallel sample serial number”. For example, the code “PV2-2” represents: the second parallel specimen (2) after 2 corrosion cycles (2) of V-groove welded joint specimen (V) in fatigue test (P). This coding system ensures traceability of test samples and scientific management of experimental data. The morphology of the finished V-groove welded joint specimens is shown in Figure 2a. Due to the limitation of polishing equipment, the production factory only polished some corroded specimens. Due to the shooting conditions of the production factory, only two specimens from each group were taken for recording. The distribution of the base metal zone, heat-affected zone, and weld zone of the V-groove welded joint specimens is shown in Figure 2b.

### 2.2. Alternate Immersion Corrosion Test

**Figure 3:**
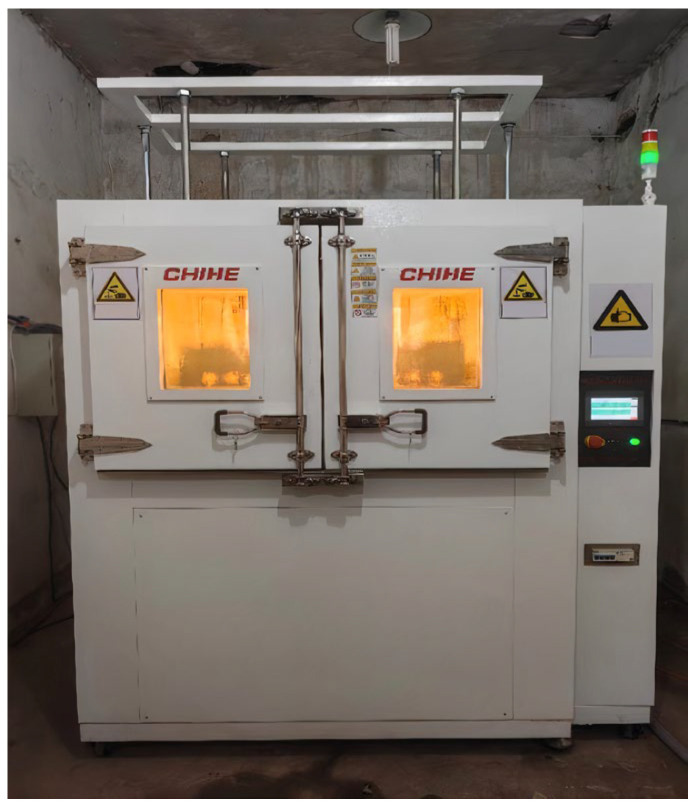
CHFL-360 (Wuxi City, Jiangsu Province, China) Cyclic immersion corrosion test chamber.

This study employed a standardized periodic immersion corrosion test apparatus (Figure 3) to conduct cyclic immersion corrosion tests on three groups of fatigue specimens (nine in total) under three corrosion durations: 0 days (0 d), 9 days (9 d), and 18 days (18 d). The corrosion process was designed in accordance with: TB/T 2375-1993 [22], and GB/T 19746-2018 [23].

When simulating the corrosive environment of plateau regions using a sodium bisulfite (NaHSO_3_) solution, periodic immersion corrosion tests were performed on weathering steel specimens via a dry-wet cyclic immersion method.

The requirements of TB/T 2375-1993 [22] were followed: when conducting periodic immersion corrosion tests via the dry-wet cyclic immersion method, the duration of each dry-wet cycle (including drying time and immersion time) was set to 60 ± 3 min—with the immersion time at 12 ± 1.5 min and the drying time at 48 ± 1.5 min [24]. During the corrosion test, the temperature in the cyclic immersion corrosion test chamber (Figure 3) was maintained at 45 ± 2 °C, and the relative humidity was maintained at 70 ± 5% RH.

**Figure 4:**
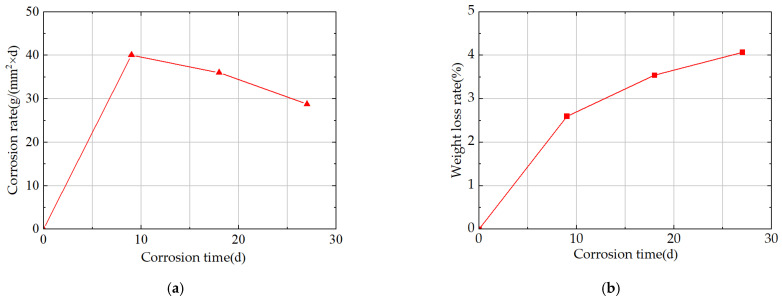
(**a**) Corrosion Rate of Q500qENH Weathering Steel V-Groove Welded Joint Specimens. (**b**) Weight loss rate of Q500qENH Weathering Steel V-Groove Welded Joint Specimens.

In related work from the same group [25], cyclic immersion corrosion tests were conducted on Q500qENH weathering steel V-groove welded joint specimens using a sodium bisulfite (NaHSO_3_) corrosion solution for durations of 0 days (0 d), 9 days (9 d), 18 days (18 d), and 27 days (27 d), and the corrosion rate of the corroded specimens was analyzed (see Figure 4a).

Results show that the specimens’ corrosion rate peaks at 9 d; from 9 d to 27 d, the corrosion rate decreases with prolonged corrosion time and gradually stabilizes. Specifically, the corrosion rate at 18 d is 10.18% lower than that at 9 d, and the corrosion rate at 27 d is 20.00% lower than that at 9 d.

The variation in weight loss rate with corrosion time in integral welded joint specimens is shown in Figure 4b. For these integral specimens, the weight loss rate reaches 2.60% at 9 d; at this point, the rust layer on the specimens remains incompletely dense, leading to a relatively high corrosion rate and a nearly linear increase in mass loss. The weight loss rate of the integral specimens reaches 3.54% at 18 d, an increase of 0.94% compared with that at 9 d; at 27 d, it reaches 4.07%, with an increase of only 0.53% compared with that at 18 d.

This phenomenon can be explained as follows: as a dense protective rust layer gradually forms on the specimen surface, the contact area between the corrosive medium and the specimen surface decreases gradually, causing the corrosion rate to decline over time and ultimately resulting in a sublinear growth characteristic of weight loss rate with corrosion time.

For exploratory analysis, the 0–18 d period in cyclic immersion corrosion tests was treated as a complete corrosion cycle; the 0–9 d interval within this cycle was defined as the “early corrosion stage,” and 9–18 d was defined as the “late corrosion stage.” Such cyclic immersion corrosion tests were conducted to explore the variation law of fatigue performance of weathering steel V-groove welded joint specimens with corrosion time.

In SO_2_-containing industrial atmospheres, weathering steel surfaces form corrosion products mainly consisting of α-FeOOH, γ-FeOOH, and small amounts of Fe_3_O_4_. The rust layer initially forms with γ-FeOOH as the initial corrosion product. As corrosion proceeds, γ-FeOOH transforms into the more stable α-FeOOH, and the oxide film densifies [26].

In related work from the same group [25], NaHSO_3_ solution was used as the corrosive medium to conduct cyclic immersion corrosion tests on Q500qENH weathering steel. XRD phase analysis of the rust layer was performed to reveal specific components: acicular γ-FeOOH, rose petal-like β-FeOOH, cotton ball-shaped α-FeOOH, and small amounts of Fe_3_O_4_. As corrosion time extends, γ-FeOOH converts to α-FeOOH. This similarity in phase evolution confirms that using NaHSO_3_ solution in cyclic immersion tests simulates corrosion processes analogous to those in SO_2_-containing plateau atmospheric environments.

Given the increasingly severe industrial pollution in the western plateau regions of China, such as the continuous rise in sulfur dioxide (SO_2_) emissions in Sichuan and Qinghai provinces [27], it is recognized that harsh environmental conditions significantly reduce the corrosion resistance of weathering steel welded joint specimens. Accelerated tests under simulated harsh conditions were therefore performed. Thus, sodium bisulfite (NaHSO_3_) solution with an initial concentration of (1.00 ± 0.05) × 10^−2^ mol/L was used as the corrosive medium. Supplementary NaHSO_3_ corrosive solution (concentration: 2.00 × 10^−2^ mol/L) was added daily, no more than twice, with each addition taking no more than two minutes to maintain the concentration of NaHSO_3_ solution in the periodic immersion corrosion test chamber at (1.00 ± 0.05) × 10^−2^ mol/L, and accelerated corrosion tests were performed on weathering steel fatigue specimens.

The concentration design is based on the following scientific rationale: firstly, the NaHSO_3_ solution can form an effective electrochemical corrosion system with the weathering steel component, significantly accelerating the corrosion process; secondly, experimental measurements show that the pH value of this NaHSO_3_ solution is 4.60 ± 0.20, which aligns well with the weakly acidic condensate environment (primarily composed of SO_2_ hydration products) naturally formed on weathering steel component surfaces in plateau regions. This experimental design not only ensures that the electrochemical characteristics of the corrosion process match real-world conditions but also guarantees the similarity in morphology and composition of corrosion products. It thereby provides a reliable experimental foundation for studying the corrosion mechanisms of weathering steel in sulfur-polluted atmospheric environments in plateau areas.

It should be clarified that the applicability of the cyclic immersion corrosion tests conducted in this study—which use (1.00 ± 0.05) × 10^−2^ mol/L NaHSO_3_ solution as the corrosion medium—is limited to highly SO_2_-enriched regions of western China (e.g., western Sichuan, eastern Qinghai).

The tests provide reference data for the corrosion behavior of 16 mm thick Q500qENH weathering steel V-groove welded joints in these regions; the corrosion morphology and rate trends they exhibit preliminarily reflect the long-term outdoor exposure characteristics of the material under such specific conditions.

It should be emphasized that these test results do not directly equate to the on-site corrosion behavior of Q500qENH weathering steel in all plateau regions or under all structural forms of components. In specific engineering applications, it is necessary to combine actual environmental parameters of the target region (e.g., SO_2_ concentration, temperature, humidity) with structural characteristics of the components and conduct further targeted verification.

### 2.3. Fatigue Testing Protocol for Welded Specimens

Multiple parameters (e.g., corrosion time, stress level, temperature) influence the fatigue performance of Q500qENH weathering steel V-groove welded joint specimens. Therefore, these three parameters are included in the fatigue test protocol for Q500qENH weathering steel V-groove welded joint specimens.

For multi-factor coupling test design, the variable control approach by Mehdi et al. [28] is referred to: gradient levels of key parameters (e.g., magnetic field intensity, purification time) are set to exploratively screen for optimal working conditions, providing references for the rational design of the “corrosion-low temperature-stress” multi-variables in fatigue tests of weathering steel. Herein, the orthogonal test design approach matches the requirements of multi-factor coupling test design. Given limited experimental resources, the L_9_(3^3^) orthogonal experimental design is used to conduct exploratory analysis of key factors influencing the fatigue performance of specimens, evaluate the main effects of each parameter on fatigue life within a feasible experimental scope, and provide direction for subsequent confirmatory experiments.

This study focuses on the fatigue performance of weathering steel V-groove welded joint specimens and examines three primary influencing factors: corrosion duration (D), ambient temperature (T), and stress level (S). A three-factor, three-level test protocol is developed using the orthogonal experimental design method. The parameter levels of the three influencing factors (D, T, S) are presented in Table 4; this design enables systematic investigation of fatigue performance under coupled environmental and mechanical conditions.

The stress level (S) is defined as the maximum applied stress value in fatigue tests. Its three parameter levels are set as 0.30×, 0.40×, and 0.50× the yield strength. The yield strength data are taken from the same research group’s literature [25], which comes from tensile tests at room temperature on 16 mm thick Q500qENH weathering steel V-groove welded joint specimens; the average yield strength of uncorroded specimens is 580.07 MPa (Table 5). A stress ratio (R) of 0.10 is maintained during the fatigue tests.

In Table 5, E is used to denote elastic modulus; *f_y_* for yield strength; *f_u_* for ultimate tensile strength; A for percentage elongation after fracture; *f_y_*/*f_u_* for yield-to-ultimate strength ratio; and εu for ultimate strain.

**Figure 5:**
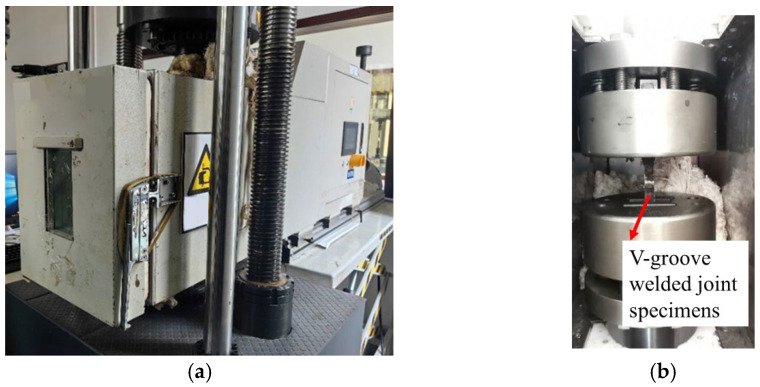
(**a**) Apparatus for low-temperature fatigue tests. (**b**) GPS-200 Fatigue testing clamp.

The single-point fatigue testing method was adopted in this study, which is defined as testing one specimen per stress level. This fatigue testing method was combined with orthogonal experimental design to efficiently utilize limited specimens. A CIMACH GPS-200 (Changchun City, Jilin Province, China) high-frequency tensile-compressive fatigue testing machine equipped with a low-temperature environmental chamber and a liquid nitrogen tank was used to systematically evaluate the cryogenic fatigue performance of the specimens (Figure 5). The tests strictly simulated two typical cryogenic conditions (−20 °C and −40 °C), with loading procedures performed according to the experimental scheme outlined in Table 4. Two failure criteria were implemented as termination conditions: when the cycle count reached 2.00 × 10^6^ cycles (i.e., two million cycles), or when the relative value of the specimen’s elongation amount attained 3 mm. Through these experiments, complete fatigue life data for all specimens were successfully obtained (detailed in Table 6), providing a reliable comprehensive dataset for analyzing fatigue performance under cryogenic conditions. Each group of specimens was subjected to the specific test once.

As shown in Table 6, some specimens did not fracture even after 2.00 × 10^6^ loading cycles, which is primarily attributed to the lower applied stress levels and the beneficial effects of low-temperature conditions on the material’s fatigue performance. For the weathering steel V-groove welded joint specimens that did not fully fracture, the fatigue loading was continued in the low-temperature fatigue testing apparatus under the same loading conditions as in the orthogonal experimental design until fracture occurred. Subsequently, the crack propagation morphology at the fracture surfaces of the specimens was characterized microscopically using a FEI Qunata600F (Hillsboro, OR, USA) scanning electron microscope (SEM).

The fatigue loading cycles of Specimen PV0-1 (T = 20 °C, S = 174 MPa) are reported as 1,319,500 cycles—fracturing before reaching the test termination cycle of 2.00 × 10^6^ cycles. The fatigue loading cycles of Specimen PV1-1 (T = −40 °C, S = 174 MPa) are noted to reach the test termination cycle (2.00 × 10^6^ cycles, no fracture), with the fatigue loading cycles increasing by ≥51.6% compared to the uncorroded group (PV0-1). Specimen PV2-2 (T = −20 °C, S = 174 MPa) is also confirmed to reach the test termination cycle (2.00 × 10^6^ cycles). Comparison of these results confirms that under the matched stress of 174 MPa, mild/moderate corrosion (9 d, 18 d) significantly extends the fatigue loading cycles compared to no corrosion.

The fatigue loading cycles of Specimen PV0-2 (T = −20 °C, S = 232 MPa) are recorded as only 332,700 cycles. Specimen PV1-2 (T = 20 °C, S = 232 MPa) is confirmed to reach the test termination cycle (2.00 × 10^6^ cycles), with its fatigue loading cycles increasing by ≥501% compared to the uncorroded group (PV0-2).

## 3. Orthogonal Experimental Analysis of Fatigue Life in Welded Specimens

Fatigue test results were analyzed using the L_9_(3^3^) orthogonal experimental design, which incorporates range analysis and variance analysis as an exploratory approach. Orthogonal arrays were used to perform mathematical and statistical analysis of multiple influencing factors, and key factors affecting the fatigue performance of welded joints were preliminarily screened. The order of priority of the three influencing factors was determined from the orthogonal experimental design results, providing direction for subsequent confirmatory experiments.

### 3.1. Extreme Value Analysis Method

During the exploratory analysis, the range *R_k_* for each influencing factor in the low-temperature fatigue test results (Table 7) was calculated to determine the optimal level combination of experimental factors.

(1) $$R_{k}=\max[{\bar{y}}_{k1},{\bar{y}}_{k2},\ldots ]-\min[{\bar{y}}_{k1},{\bar{y}}_{k2},\ldots ]$$

In the equation, (*R_k_*) represents the range of the (*k*)-th factor, indicating the variation amplitude of fatigue life when the (*k*)-th factor changes. A larger *R_k_* suggests that the factor has a greater influence on fatigue life and higher importance. And (${\bar{y}}_{kn}$) denotes the average value of the experimental factor corresponding to the (*n*)-th level of the (*k*)-th factor, which is used to determine the optimal level combination for the (*k*)-th factor. The experimental results demonstrate a positive correlation between the parameter ${\bar{y}}_{kn}$ and the fatigue performance of specimens, where higher ${\bar{y}}_{kn}$ values indicate superior fatigue resistance. As shown in Table 7, the maximum average values of the experimental index for influencing factors D, T, and S are ${\bar{y}}_{D2}$, ${\bar{y}}_{T3}$, and ${\bar{y}}_{S1}$, respectively. Consequently, it can be inferred that the combination D_2_T_3_S_1_ represents the most favorable factor configuration for enhancing the fatigue performance of the specimens.

Analysis of fatigue performance test results (Table 7) revealed that the calculated influence factors ranked as *R*_S_ > *R*_D_ > *R*_T_ according to Formula (1), indicating distinct differences in the influence of each parameter on fatigue life of V-groove welded joint specimens. The research demonstrates that Q500qENH weathering steel V-groove welded joint specimens exhibited optimal fatigue performance under the following conditions: 9 d corrosion duration, −40 °C test temperature, and 174 MPa stress level. Parameter sensitivity analysis identified stress level (*R*_S_) as the most influential factor on fatigue life, followed by corrosion duration (*R*_D_), while environmental temperature (*R*_T_) showed relatively minor effects. The initiation of metal fatigue cracks is affected by multiple factors, including alternating loads, surface conditions (such as surface defects and reduction in effective cross-sectional size), and environmental factors (such as temperature). Therefore, changes in the fatigue loading stress level and the gradual erosion of the specimen’s effective cross-section by the corrosive solution will directly affect the stress state of the specimen, ultimately altering its ability to resist crack propagation. Metals have a brittle-ductile transition temperature line. In the fatigue test, the temperature conditions vary within the range of the brittle-ductile transition temperature line, and the fluctuation of temperature conditions is small, which has little impact on the material properties of the specimen itself (such as brittleness and ductility) and is difficult to change the specimen’s ability to resist crack propagation. Hence, the influence of the temperature conditions adopted in the test on the fatigue performance of the weathering steel V-groove welded joint specimens is lower than that of the loading stress level and corrosion time. These findings provide critical insights for parameter optimization of weathering steel welded structures in corrosion-fatigue environments, offering particularly valuable guidance for the design of welded structures subjected to alternating loads, such as railway vehicles and bridges.

### 3.2. Analysis of Variance

The analysis of variance method was used to process low-temperature fatigue test data to verify the accuracy of range analysis results. In the analysis of variance method, 9 specimens have a total of 8 degrees of freedom. The degrees of freedom (d*f*) for corrosion duration (D), temperature (T), stress level (S), and error (e) were allocated in a balanced manner (see Table 8) to ensure no remaining degrees of freedom and meet the conditions of a saturated design.

The specimen test results (Table 7) were used to calculate the sum of squared deviations (SS), degrees of freedom (d*f*), mean squared error (MSE), and total sum of squared deviations (F*_k_*) for the influencing factors (D), (T), (S), and the error term (e) [29,30]. The results of the sum of squared deviations (SS) and total sum of squared deviations (F*_k_*) were compared, and an exploratory analysis was conducted on the key factors influencing the fatigue performance of Q500qENH weathering steel V-groove welded joint specimens.

The calculation formulas for the sum of squared deviations (SS) and mean squared error (MSE) are as follows:

(2) $${\mathrm{SS}}_{k}=\frac{a}{m}{\sum_{n=1}^{m}\left({\bar{y}}_{kn}\times 3\right)}^{2}-\frac{1}{m}{\left(\sum_{n=1}^{m}\left({\bar{y}}_{kn}\times 3\right)\right)}^{2}$$

(3) $${\mathrm{MSE}}_{k}=\frac{{\mathrm{SS}}_{k}}{df}$$

(4) $$F_{k}=\frac{{\mathrm{MSE}}_{k}}{{\mathrm{MSE}}_{e}}$$

In the formula, “*a*” represents the total number of influencing factors in the orthogonal test; “*m*” represents the number of orthogonal test runs.

For the quantitative analysis of Table 8 results, stress level is confirmed to exert the strongest regulatory effect on fatigue life (F_S_ = 6.07, accounting for 66.97% of the total SS), followed by corrosion duration (F_D_ = 1.61, accounting for 17.77% of the total SS), and ambient temperature exerts the weakest effect (F_T_ = 0.39, accounting for 4.26% of the total SS). These results align with the range analysis (R*_k_*: S = 1,566,800 > D = 804,700 > T = 389,700), further verifying the primary and secondary order of factor influences (Table 8).

The influence contribution degree of each factor on fatigue life was quantified using the sum of squared deviations (SS) and F-statistic: for stress level (S), SS = 36.90 × 10^11^ and F_S_ = 6.07; for corrosion duration (D), SS = 9.79 × 10^11^ and F_D_ = 1.61; for ambient temperature (T), SS = 2.35 × 10^11^ and F_T_ = 0.39. These values show the order of influence magnitude as S > D > T.

Exploratory range and variance analyses were conducted on the orthogonal experiment results, determining the primary and secondary order of factors affecting the steel’s fatigue life as follows: stress level (S, R_S_, with the highest influence contribution degree) > corrosion duration (D, R_D_) > ambient temperature (T, R_T_, with the lowest influence contribution degree).

Multifactor interaction analysis was conducted, confirming that under low-temperature conditions (−40 °C), extending the corrosion duration to 9 days combined with a lower stress level of 174 MPa yields the optimal fatigue performance for Q500qENH weathering steel V-groove welded joints, with the fatigue lifespan of the joints being significantly higher than that of other test groups.

This phenomenon is interpreted by the following mechanisms: first, a lower stress level (S, R_S_) contributes to improving fatigue life to a certain extent; second, a shorter corrosion duration (D, R_D_) helps remove initial defects on the specimen surface and enhance specimen integrity; finally, low temperature (T, R_T_) induces internal metal atoms of the specimen to enter a low-energy state, increases the energy required for metal atom migration, and thereby inhibits crack propagation. The synergistic effect of these three factors effectively enhances the material’s fatigue resistance.

## 4. Validation of Welding Finite Element Models

To obtain a comprehensive full-life analysis of fatigue crack initiation, propagation, and fracture, macroscopic and microscopic fractographic analysis were conducted on the fatigue fracture surfaces of the specimens based on fatigue crack propagation theory and scanning electron microscopy (SEM). For correlative analysis between microstructure and macroscopic properties, the multi-characterization combination method by Mehdi et al. [31] is referred to: techniques such as SEM, XRD, and Raman are used to quantify the material’s microstructural characteristics (e.g., MXene interlayer spacing, defect density), the correlation between these characteristics and macroscopic properties (e.g., electrochemical stability) is then established, providing methodological references for the mechanism analysis of “rust layer structure–welding defects–fatigue life” in weathering steel.

**Figure 6:**
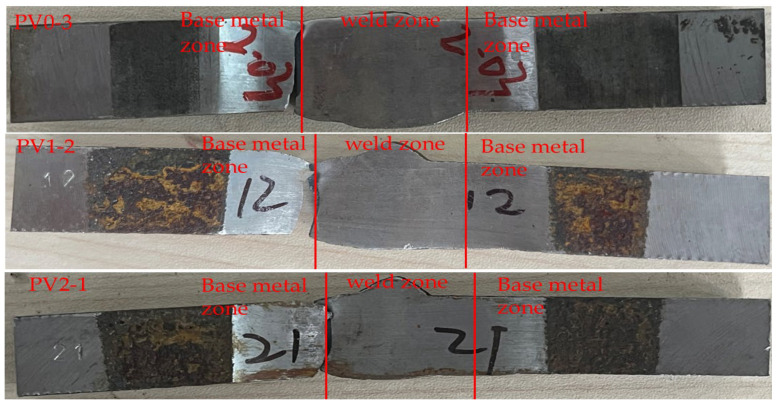
Location of specimen fracture surface.

**Figure 7:**
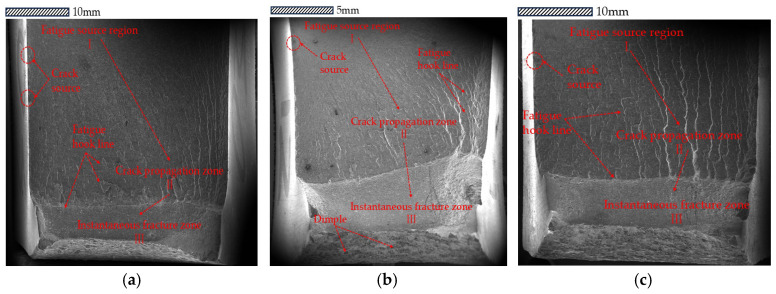
(**a**) Macroscopic morphology of fracture surface of PV0-3 fatigue specimens. (**b**) Macroscopic morphology of fracture surface of PV1-2 fatigue specimens. (**c**) Macroscopic morphology of fracture surface of PV2-1 fatigue specimens.

The results of the orthogonal experimental design were analyzed, finding that ambient temperature has the lowest influence contribution degree on fatigue life, while stress level has the highest. Thus, analysis focuses on the fracture locations and macroscopic fracture surfaces of fatigue specimens with three different corrosion durations (0 d, 9 d, 18 d) under a stress level of 290 MPa, with the relevant results presented in Figure 6 and Figure 7.

An exploratory study was conducted on the crack propagation mechanisms and fracture morphologies in the three primary regions of fatigue fracture (fatigue initiation zone, crack propagation zone, instantaneous fracture zone) to validate the intrinsic mechanisms of fatigue fracture.

### 4.1. Macrofractographic Analysis of Fatigue Fracture Surfaces

The fatigue crack behavior of corroded weathering steel V-groove welded joint specimens is analyzed by combining the fatigue fracture theory of welded specimens and findings of corrosion morphology scanning.

**Figure 8:**
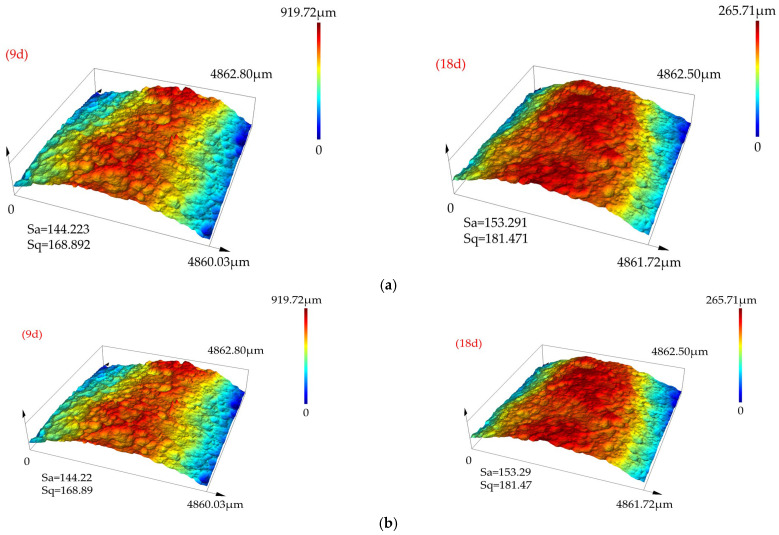
(**a**) Corrosion morphology of weld zone (WZ) in weathering steel V-groove welded joint specimens. (**b**) Corrosion morphology of base metal zone (BWZ) in weathering steel V-groove welded joint specimens.

In related work from the same group [32], the 3D surface roughness of corroded weathering steel V-groove welded joint specimens was obtained using a 3D laser scanner (as presented in Figure 8). An exploratory analysis of the influence mechanism of the corrosion factor on specimen crack initiation is conducted by combining the macroscopic fracture morphology of weathering steel V-groove welded joint specimens (as presented in Figure 7), and the crack initiation location is determined.

It is observed that fatigue cracks initiate at the weld toe-base metal interface, with crack sources near the specimen surface. This phenomenon is attributed to abrupt cross-sectional transitions at the weld–base metal interface—geometric discontinuity at the specimen’s weld causes stress concentration at the weld toe-base metal interface.

For uncorroded specimens, their fracture surfaces are relatively smooth and flat, with fatigue source areas uniformly distributed. For V-groove welded joint specimens corroded for 9 days (9 d), surface pitting and fatigue cracks are observed; this phenomenon is attributed to pitting forming microscopic stress concentration points, which further induce crack initiation.

For specimens corroded for 18 days (18 d), it is observed that as corrosion time extends, the pitting pit density on the specimen surface increases (as presented in Figure 8), and numerous obvious fatigue cracks exist in the crack propagation zone. When compared with the fracture morphology of specimens corroded for 9 d, synergistic effects between adjacent pits are observed—multiple pits act as simultaneous crack sources to jointly promote crack initiation. This indicates that as corrosion time extends, the specimen’s crack propagation behavior has shifted from “single initiation” to “multi-crack synergistic propagation”.

The propagation path of fatigue cracks is traced back to clarify their complete evolution process. It is observed that cracks first gradually penetrate along the transverse width of the specimen surface, then extend longitudinally along the specimen thickness direction until the specimens fracture. This propagation process is correlated with the coupling effect of continuous corrosion pitting evolution and cyclic loading to clarify the driving mechanism of crack path evolution.

### 4.2. Scanning Electron Microscopy (SEM) Fractographic Analysis of Fatigue Fracture Surfaces

To further investigate the mechanisms of crack initiation and propagation, a 10 mm cross-section near the fracture surface of the V-groove welded joint specimen was extracted using wire cutting. Subsequently, the fracture morphology of the specimen was characterized by Scanning Electron Microscopy (SEM) to elucidate the microstructural evolution during crack propagation under different corrosion cycles.

**Figure 9:**
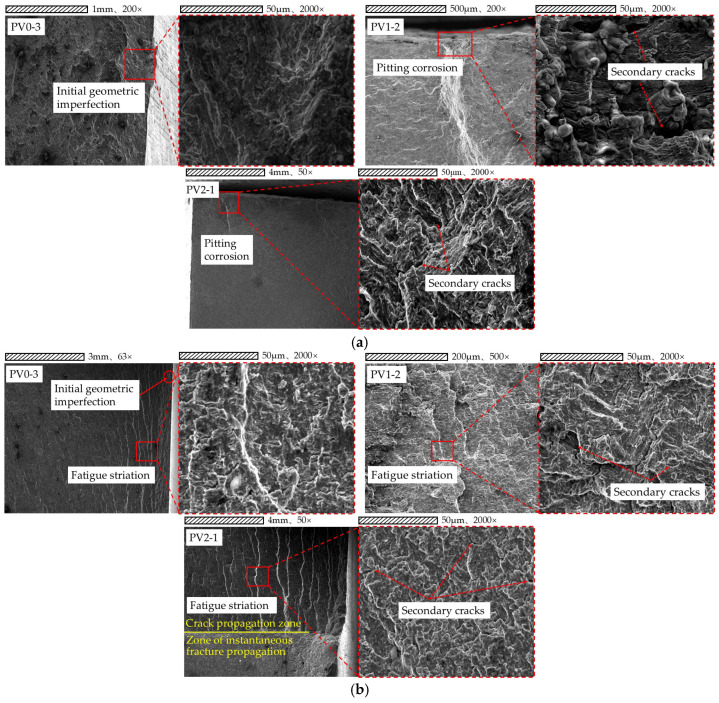
(**a**) Fatigue source region of PV0-3, PV1-2, and PV2-1 specimens. (**b**) Crack propagation zone of PV0-3, PV1-2, and PV2-1 specimens.

Scanning electron microscope (SEM) micrographs (as presented in Figure 9) are observed, and the distinct fracture characteristics are analyzed; an exploratory study is conducted on the transition process of cracks from initiation to stable growth, as well as the interaction between corrosion-induced defects and crack trajectories.

The scanning morphologies of corrosion pits and initial manufacturing defects (e.g., slag inclusions, gas pores) are compared: corrosion pits mostly appear as irregular depressions with corrosion products along their edges, while initial manufacturing defects mostly exhibit linear or tear-like traces.

Fatigue crack growth theory is applied to conduct macroscopic observations on the fracture surfaces of fatigue specimens, and it is confirmed that fatigue cracks typically initiate at stress concentration zones. Microscopic observations are performed, finding that numerous irregular fatigue striations exist in crack initiation zones, and secondary cracks associated with crack initiation are distributed on fracture surfaces—with the width direction of these secondary cracks consistent with the crack propagation direction.

The fatigue crack propagation origins of the specimens are traced, finding that fatigue cracks in uncorroded V-groove welded joints typically initiate at internal manufacturing defects such as slag inclusions and gas pores; in contrast, fatigue cracks in corroded V-groove welded joint specimens predominantly initiate at corrosion pits and tear ridges. Microstructural analysis shows that the presence of corrosion pits causes stress concentration, which not only accelerates the crack initiation process but also reduces the resistance to corrosion-fatigue crack initiation and propagation in the pitted regions of the material. With prolonged corrosion exposure time, the increasing density of corrosion pits led to synergistic interactions between adjacent pits, thereby accelerating fatigue failure. The corrosion pits acted as critical stress concentrators that significantly reduced the fatigue life of welded joints by providing preferential sites for fatigue crack initiation and facilitating multi-site damage accumulation under cyclic loading conditions.

The microscopic morphology of fracture surfaces is analyzed, with typical fatigue fracture characteristics observed during crack propagation. In the crack propagation zone, it is identified that the metallic material at the crack tip undergoes significant plastic deformation and tearing, with the tearing direction perpendicular to the crack growth direction. When fatigue cracks propagate within single grains, their directional stability is observed; this stability is indicated to cause the material to form a series of parallel fatigue striations under cyclic stress.

The fatigue crack propagation process (as presented in Figure 9) is analyzed, confirming that fatigue cracks mainly initiate at stress concentration zones. V-groove welded joint specimens are observed using a scanning electron microscope (SEM), with irregular fatigue striations and secondary cracks aligned with the propagation direction identified in their crack origin regions. An investigation is conducted to determine two typical crack initiation modes: cracks in uncorroded specimens originate from internal defects (slag inclusions/gas pores), while cracks in corroded specimens originate from corrosion pits and tear ridges. Since the irregular geometry of corrosion pits generates significant stress concentrations—and the synergistic effect of these stress concentrations intensifies with prolonged corrosion exposure—corrosion pits become a critical factor accelerating crack initiation. It is recognized that the crack propagation zone exhibits features of polycrystalline materials, including orthogonal tear marks and multi-oriented fatigue striations; these features reflect the complex propagation paths induced by differences in grain orientation.

The above findings are synthesized to elucidate: as stress concentration sources, corrosion pits significantly reduce the fatigue life of welded joints by promoting multi-source damage accumulation. This conclusion provides important insights for exploring the fatigue failure mechanism of weathering steel welded joints under environmental-mechanical coupling effects.

## 5. Finite Element Modeling and Analysis of Welding Processes

The combined analytical approach based on ABAQUS (Version 2021) and FRANC 3D (Version 7.0) software is used to simulate the fatigue performance changes in weathering steel specimens affected by corrosion under low-temperature conditions.

First, fatigue performance analysis is conducted on the “PV0-3” specimen under the condition of maximum stress of 0.50 *f_y_* (where *f_y_* denotes yield strength, corresponding to 290 MPa). Then, the obtained fatigue life results of the model are compared with the fatigue test results of the specimen to verify the model’s effectiveness. Finally, fatigue performance simulation is performed on the weathering steel specimen model under low-temperature conditions at different maximum stress levels.

An exploratory analysis is conducted on the influence mechanism of key factors on the fatigue performance of weathering steel V-groove welded joint specimens.

### 5.1. Model Parameter Optimization and Design

**Figure 10:**
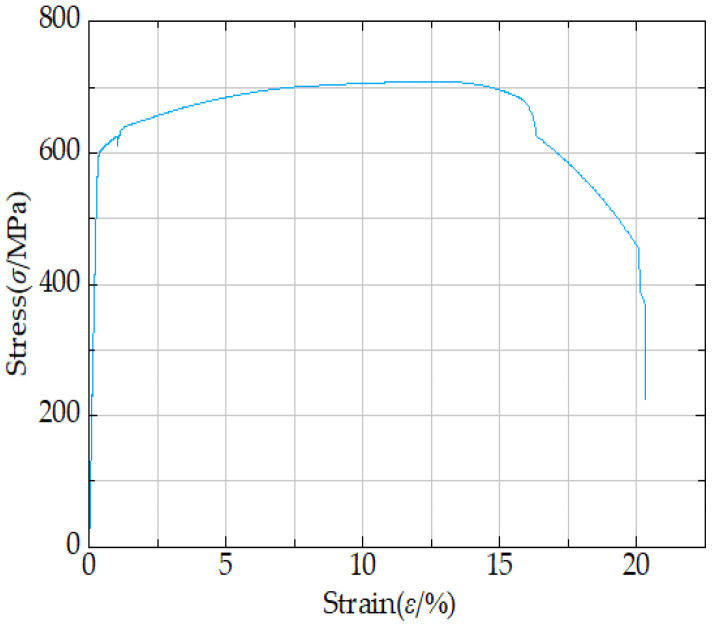
Stress–strain curve of weathering steel V-groove welded joint specimens at −40 °C.

To more accurately reflect the material properties of the actual weathering steel specimens, establish the specimen model dimensions using the experimental specimen dimensions. Using the linear elastic fracture mechanics approach, incorporate only the elastic phase parameters of the material into the specimen model simulation. Table 9 is taken from the same research group’s literature [33], which contains the measured crack growth values of Q500qENH steel at −40 °C. T represents the ambient temperature; E denotes the elastic modulus; *ρ* stands for the density; *μ* indicates Poisson’s ratio; C and m are the crack growth parameters in the Paris formula; and A refers to the percentage elongation after fracture. Figure 10 presents the stress–strain curve of weathering steel V-groove welded joint specimens at −40 °C.

Analyzing the scanning results of corrosion roughness for weathering steel V-groove welded joint specimens shows that most surface corrosion on the specimens appears as pitting pits. These pits weakly weaken the effective cross-section of the specimens. Thus, corrosion’s influence on the specimens’ elastic properties is not considered, and the elastic modulus remains unadjusted.

### 5.2. Static Analysis of Fatigue Specimen Model

To accurately simulate the mechanical response of fatigue specimens under actual service conditions, first, the geometric dimensions of weathering steel V-groove welded joint specimens are referred to, and a finite element model is established in ABAQUS software; detailed static stress analysis is then conducted on the specimens. Next, the specimen’s finite element model is imported into FRANC 3D software, where it is divided into submodels. An initial crack is set in the submodel within FRANC 3D.

In this study, small specimens are used, and GB 50661-2011 [20] is strictly followed during the welding process; additionally, the weld zone is ground and polished after welding—these measures reduce the influence of welding residual stress on the specimens. Furthermore, this study aims to conduct an exploratory analysis of the key factors affecting the fatigue performance of V-groove welded joint specimens of Q500qENH weathering steel. The fatigue life results from finite element simulation are compared with those from fatigue tests; the error between the two does not exceed 10%. Thus, the influence of welding residual stress is not considered in the finite element simulation of this study.

As shown in the Paris Formula (5), the sensitivity of the finite element model to the Paris constants is expressed as follows: as C and m increase, the fatigue life of the finite element model decreases gradually.

dN = da/(*C*(Δ*K*)*^m^*) (5)

**Figure 11:**
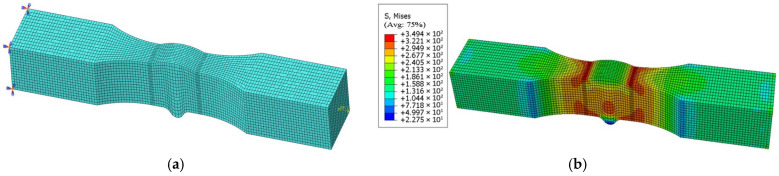
(**a**) Mesh generation of PV0-3 specimen. (**b**) Static analysis of PV0-3 specimen.

Paris parameters are assigned to the submodel (as presented in Table 9), and crack propagation simulation is then performed. When the finite element model is established, boundary conditions where one end of the specimen model is fixed and the other end bears an axial equivalent concentrated load are adopted. The concentrated load value is taken as 0.50 times the yield strength (0.50 × 580.07 MPa) from Table 5, and the equivalent concentrated load (46,400 N) applied to the boundary of the specimen model is derived through cross-sectional dimension conversion. The mesh size of the specimen model for calculation is set to 1 mm (Figure 11a).

**Figure 12:**
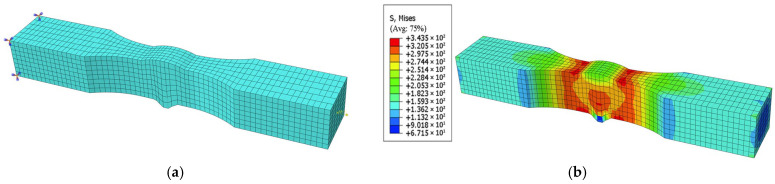

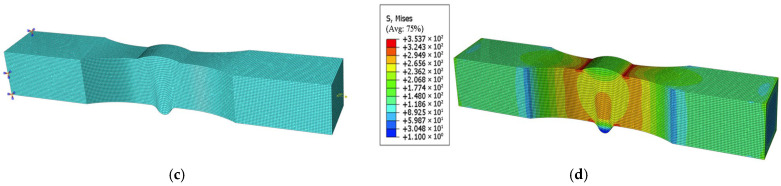
(**a**) Model of PV0-3 specimen with a mesh size of 2.00 mm. (**b**) Stress results of PV0-3 specimen model with a mesh size of 2.00 mm. (**c**) Model of PV0-3 specimen with a mesh size of 0.50 mm. (**d**) Stress results of PV0-3 specimen model with a mesh size of 0.50 mm.

Considering that mesh sensitivity affects the results of the specimen model, the stress nephogram of the calculation specimen model (Figure 11b) is compared with those of specimen models with mesh sizes of 2.00 mm (Figure 12b) and 0.50 mm (Figure 12d). The final results show that the stress result of the coarse-mesh model deviates by 1.69% from that of the calculation model (not exceeding 2.00%) and the stress result of the fine-mesh model deviates by 1.23% from that of the calculation model (also not exceeding 2.00%). It is confirmed that this result indicates the specimen model is mesh-insensitive and can achieve accurate numerical simulation.

Given that geometric discontinuities in the specimen shape tend to induce stress concentration effects, the mesh in the parallel section and weld zone of the calculation specimen model (as presented in Figure 11a) is locally refined, and the model is discretized using reduced-integration C3D8R hexahedral elements.

Numerical simulation results (Figure 11b) reveal significant stress concentration at the junction of the weld toe and base metal zone, with stress levels significantly higher than in other regions. Such stress concentration under cyclic loading may significantly reduce structural fatigue life and thus deserves special attention in engineering design.

### 5.3. Fatigue Crack Propagation Analysis in Test Specimens

#### 5.3.1. Subdivision Modeling

**Figure 13:**
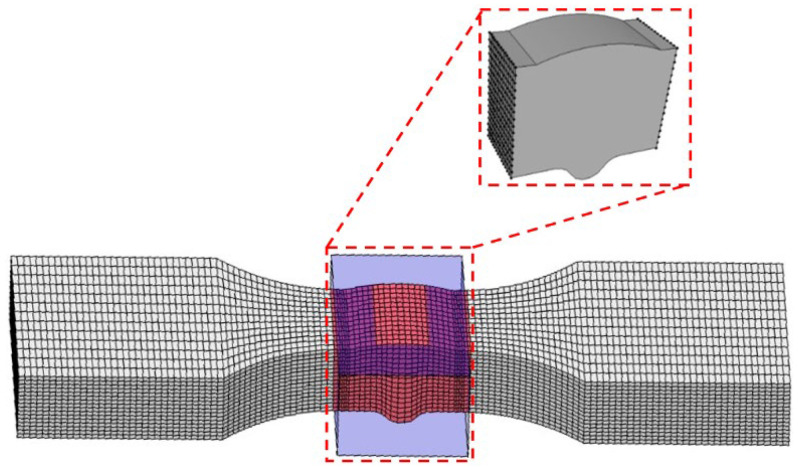
Subdivision process flowchart.

The INP file exported from the ABAQUS global model was imported into FRANC 3D software, where an initial crack was inserted at the weld toe-base metal transition zone. Subsequently, the submodel shown in Figure 13 was extracted through mesh partitioning.

#### 5.3.2. Introduction of Initial Crack

The static simulation results at −40 °C and the experimentally observed fracture locations are combined to determine the initial crack positions, and it is noted that fatigue cracks typically initiate at the junction of the weld toe and base metal. Initial cracks are introduced at the weld toe–base metal interface junction in the uncorroded model; in the corroded model, initial cracks are introduced at pitting locations near the interface of the weld toe and heat-affected zone (HAZ).

An elliptical crack configuration is adopted as the initial crack form for the V-groove welded joint specimen model. The crack dimensions are defined by the semi-minor axis length *b* (mm) and semi-major axis length *c* (mm) of the elliptical crack, and the aspect ratio is fixed at *b*/*c* = 1.

3D pitting morphology scans of weathering steel V-groove welded joint specimens corroded for 9 days and 18 days were conducted, as reported in the same research group’s literature [32]; it is observed from the scanning results that in the specimen’s base metal zone and weld zone: when the corrosion time reaches 9 days, pits gradually form a semi-elliptical shape; when the corrosion time reaches 18 days, the pit size increases significantly, dominated by local corrosion, and most pits connect with each other to form a penetrating state.

Given that most pits interconnect, it is difficult to divide hexahedral meshes accurately. To ensure effective meshing of the specimen model, the pit morphology is simplified by introducing a larger pitting pit at the weld toe-base metal junction of the specimen, thereby simplifying the simulation of crack propagation in corroded specimens. The 3D surface roughness scanning results in Figure 8 and the corrosion parameters in Table 10 are incorporated, and the pitting depth is set to 0.20 mm and the pitting width to 0.80 mm.

The linear elastic mechanics method is adopted for crack propagation simulation. The specimen’s thickness and width are combined, and with reference to the research conclusion by Zong et al. [34] (i.e., initial crack size should range from 0.10 mm to 0.50 mm), the initial crack size of the specimen model is determined to ensure the effectiveness of initial crack introduction and a sufficient crack propagation path.

With reference to the fatigue fracture analysis results of weathering steel V-groove welded joint specimens, it is noted that fatigue cracks typically occur at the junction of the base metal zone and weld toe. Therefore, the initial crack position of the model is set at the junction of the base metal zone and weld toe.

According to the specimen’s thickness, both the initial crack width and depth of the uncorroded model are set to 0.40 mm. For the corrosion model, the influence of pitting pits on crack propagation is considered: a pitting pit is introduced at the junction of the base metal zone and weld toe, and an initial crack is introduced at the bottom of the pitting pit to simulate crack initiation induced by pitting-induced stress concentration. Given that the pitting pit has a depth of 0.20 mm and a width of 0.80 mm, both the initial crack width and depth of the corrosion model are set to 0.10 mm. This approach accounts for both geometric discontinuity effects in uncorroded conditions and localized stress concentration induced by corrosion pits, while maintaining consistency with established fracture mechanics principles for fatigue crack growth analysis.

**Figure 14:**
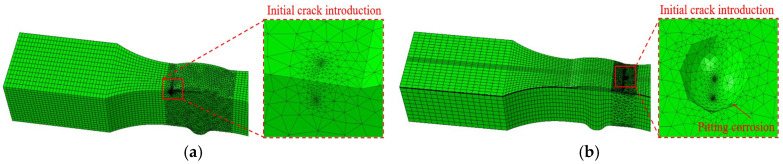
(**a**) Introduce initial cracks on the surface of the specimen model. (**b**) Initial crack initiated from the bottom of corrosion pit in specimen model.

For the uncorroded specimen model, the initial crack dimensions—where *b* denotes the semi-minor axis and *c* denotes the semi-major axis of the elliptical crack—are set with both *b* and *c* equal to 0.40 mm, and the crack front template radius is set to 10.00% of the initial crack size. The submodel mesh is then redefined (as presented in Figure 14a) to optimize mesh accuracy for subsequent crack propagation simulation.

The size of the initial crack directly affects the calculation of the stress intensity factor (SIF) and thus influences fatigue life prediction. For the Q500 weathering steel model, different initial crack sizes are introduced in the literature [33] from the same research group, including models that only vary the initial crack width *c* and models that only vary the initial crack depth *b*. Following the linear elastic fracture mechanics theory, the variation law of the model’s fatigue life is analyzed. The results show that when the initial crack width *c* increases by 0.10 mm, the model’s fatigue life decreases by an average of 1.99%; when the initial crack depth *b* increases by 0.10 mm, the model’s fatigue life decreases by an average of 3.28%.

The pit depth of the V-groove welded joint specimen in the finite element model was set to 0.20 mm, and the pit width was 0.80 mm. An initial crack with dimensions *b* = 0.10 mm and *c* = 0.10 mm was inserted at the bottom of the corrosion pit (Figure 14b).

Pit corrosion is the core inducement for crack initiation in corroded specimens. The parameters of the single pit introduced in this study are set according to the 3D roughness scanning results of the 5 mm × 5 mm area (depth = 0.20 mm, width = 0.80 mm).

A simplified analysis is conducted on specimens with pits: the width and depth of pits reduce the effective cross-sectional area of specimens, thereby decreasing their fatigue life. The single pit is treated as an approximation of the initial crack; its shape sensitivity is similar to that of the initial crack.

#### 5.3.3. Static Analysis

On the ABAQUS finite element analysis platform, static analysis of the specimen is conducted, and the interaction integral method (M-integral) is used to calculate the stress intensity factors of the specimen model. During numerical simulation, an equivalent treatment method for mixed-mode cracking is introduced to address the mixed-mode crack issue in the model. The equivalent stress intensity factor Δ*K*_e_ [32] is employed to simulate three-dimensional crack propagation and further obtain the crack propagation characteristics.

**Figure 15:**
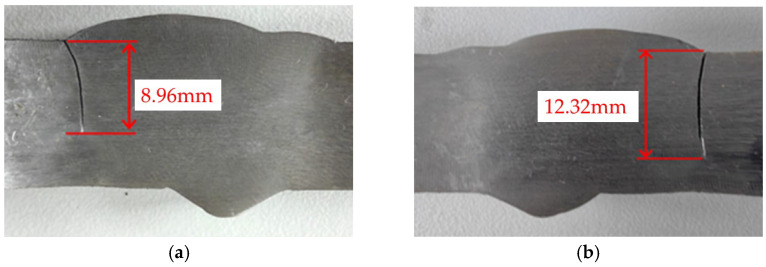
(**a**) Crack on the front surface of V-groove welded joint specimen; (**b**) Crack on the back surface of V-groove welded joint specimen.

Reference is made to the same-group literature [25], which conducts fatigue tests on 16 mm thick Q500qENH weathering steel V-groove welded joint specimens and investigates the critical value for the specimens’ fatigue failure; from which it is observed that the specimens fail when the crack size reaches 56% to 77% of the specimen thickness (as shown in Figure 15). Thus, in this study, this proportion range is taken as a reference, and 75% of the specimen thickness is adopted as the critical failure size for simulating the crack propagation process of the specimen model.

Paris’ fatigue crack propagation theory is employed, and the equivalent stress intensity factor method is incorporated to predict the fatigue life (N).

### 5.4. Fatigue Test Results Validation

**Figure 16:**
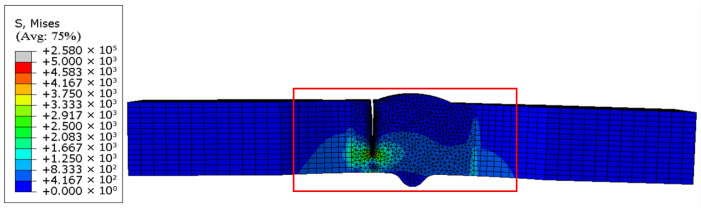
Numerical simulation of crack propagation loci in test specimens.

**Figure 17:**
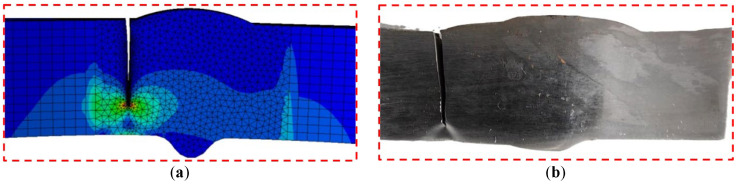
(**a**) Crack propagation simulation results of V-groove welded joint specimen model; (**b**) Crack propagation results of fatigue tests on V-groove welded joint specimens.

For the model, when its fatigue crack propagates to the failure state, the stress distribution is presented in Figure 16. The fatigue crack morphology of the failed model (Figure 17a) is compared with the fatigue test results of the specimen (Figure 17b), and it is confirmed that the crack propagation path and fracture location obtained from numerical simulation are highly consistent with the experimental observations.

The effectiveness of introducing initial cracks at the weld toe–base metal interface for fatigue crack growth simulation is validated, and it is further confirmed that this method can accurately reflect the fracture behavior characteristics of actual specimens.

## 6. Fatigue Performance Simulation of Welded Joint Models

During the analysis of orthogonal test design results, it is identified that the influence of temperature on the specimens’ fatigue life is weaker than that of corrosion duration and stress level; it is also identified that −40 °C (a low-temperature environment) is one of the conditions that enable the specimens to achieve optimal fatigue life.

To explore the effects of corrosion duration and stress level on the specimens’ fatigue life under low-temperature conditions, crack propagation simulations are conducted on weathering steel V-groove welded joint specimens under −40 °C conditions.

The simulation results (Table 11) revealed the variations in fatigue life “N” and crack propagation length “*a*” of V-groove welded joint specimens under different stress levels. Specifically, the numerical simulation results in Table 11 were obtained by constructing a three-dimensional finite element model of Q500qENH weathering steel V-groove welded joint specimens using ABAQUS software. Material parameters were adopted from Table 9. A fully fixed boundary condition was applied to one side of the model, while cyclic loading was applied to the other side, consistent with the fatigue loading setup described in the fatigue test (as shown in Figure 11). Initial cracks were introduced into the specimen model using FRANC 3D, and crack propagation simulations were performed using the Paris equation. The simulation was stopped when the crack propagation depth reached approximately 75% of the specimen thickness. Key output parameters, including stress level, fatigue life, and crack propagation depth (Table 11), were extracted. Each set of specimens underwent three independent repeated simulations. The extracted results were averaged to reduce random errors, and the final values were rounded to two significant figures.

Since the crack propagation life in the initial stage of fatigue simulation testing constitutes the predominant portion of the total fatigue life of the specimen model, the number of stress cycles required for the crack to propagate to its maximum length in the specimen model is adopted to represent its fatigue life. A comparison between the numerically simulated fatigue life results of the specimen model and the experimental fatigue life results obtained from physical testing (Table 12) reveals that the relative error between the two sets of fatigue life data falls within 10.00%. This close agreement indicates that the numerical simulation results are essentially consistent with the experimental results, thereby validating the accuracy of the crack propagation modeling in the finite element analysis.

**Figure 18:**
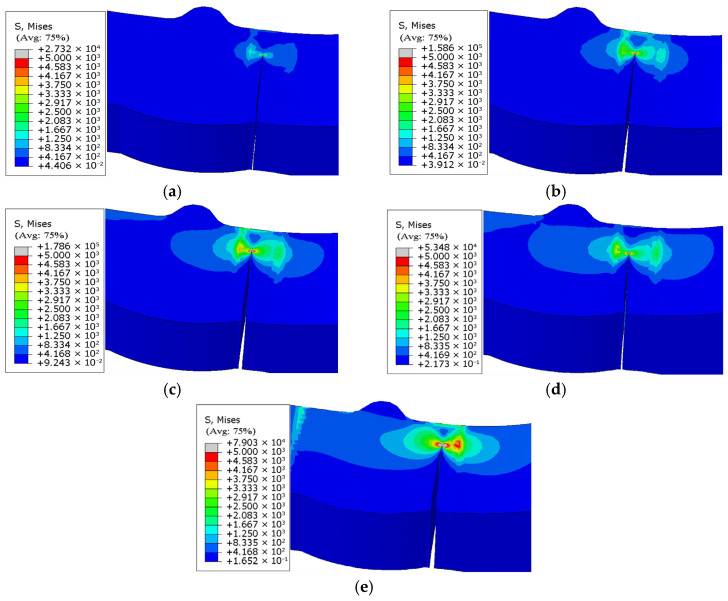
(**a**) Fracture morphology and stress cloud of uncorroded specimen under 174 MPa stress. (**b**) Fracture morphology and stress cloud of uncorroded specimen under 203 MPa stress. (**c**) Fracture morphology and stress cloud of uncorroded specimen under 232 MPa stress. (**d**) Fracture morphology and stress cloud of uncorroded specimen under 261 MPa stress. (**e**) Fracture morphology and stress cloud of uncorroded specimen under 290 MPa stress.

**Figure 19:**
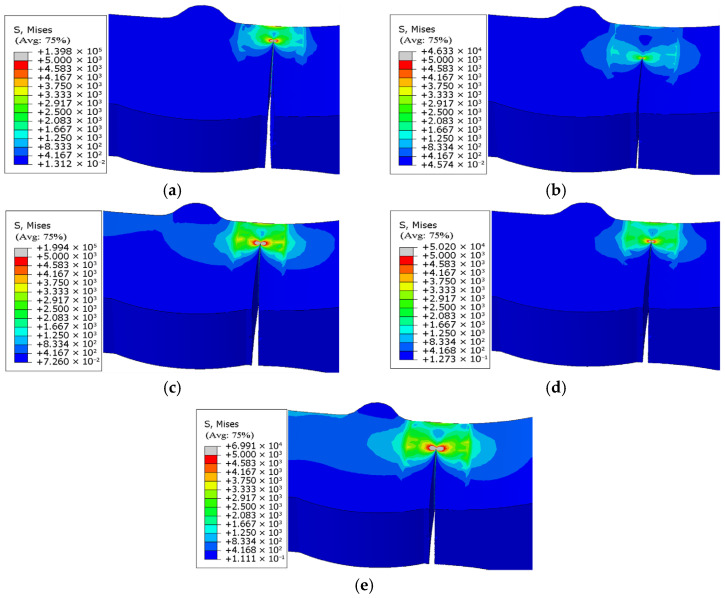
(**a**) Fracture morphology and stress cloud of corroded 18-day specimen under 174 MPa stress. (**b**) Fracture morphology and stress cloud of corroded 18-day specimen under 203 MPa stress. (**c**) Fracture morphology and stress cloud of corroded 18-day specimen under 232 MPa stress. (**d**) Fracture morphology and stress cloud of corroded 18-day specimen under 261 MPa stress. (**e**) Fracture morphology and stress cloud of corroded 18-day specimen under 290 MPa stress.

The fracture morphology and stress nephogram of the uncorroded specimen model (Figure 18) and the corroded specimen model (Figure 19) are analyzed, and it is found that the fatigue fracture of both types of specimen models occurs consistently at the junction of the weld toe and base metal interface. This characteristic is consistent with the results of low-temperature fatigue tests.

Regarding crack propagation behavior, the specimen models exhibited distinct directional characteristics: the crack width continuously expanded in the horizontal direction with increasing stress cycles, while the crack depth progressively increased in the longitudinal direction, ultimately leading to complete penetration of the model.

**Figure 20:**
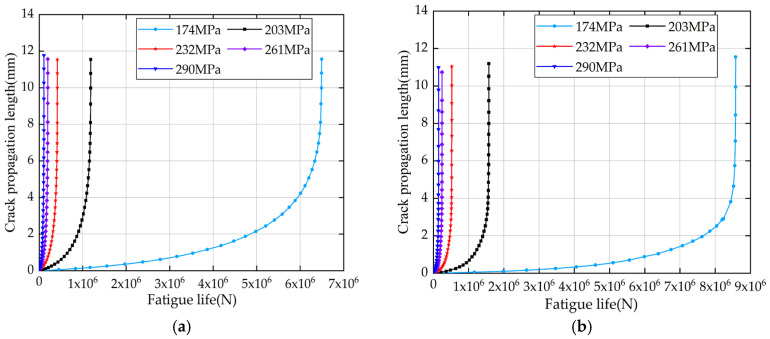
(**a**) Fatigue life curves of uncorroded specimens. (**b**) Fatigue life curves of corroded 18-day specimens.

Furthermore, based on the fatigue life results of specimen models (Table 11), crack propagation length “*a*” versus cycle number “N” curves were established for both uncorroded (Figure 18) and corroded specimen models (Figure 19), as shown in Figure 20. These curves not only verify the reliability of the specimen models’ fatigue life predictions but also provide compelling evidence for the validity of the crack propagation behavior analysis.

To ensure the reliability of the fatigue data, a detailed analysis of crack propagation behavior in V-groove welded joint specimens was conducted. As shown in Figure 20, a clear relationship between crack length (*b*) and the number of cycles (N) was established, highlighting the progression of fatigue damage under varying stress levels. This analysis focuses on the variation law of fatigue life of V-groove welded joint specimens of the new Q500qENH weathering steel after being affected by corrosion. The results demonstrate that under non-corroded conditions, the fatigue life of the specimen model exhibits a significant decreasing trend with increasing stress levels (Figure 20a). Meanwhile, the crack growth rate continuously rises with increasing crack length, manifested as a monotonically increasing slope of the curve. As the specimen approaches failure, the curve slope tends toward infinity, indicating that the crack propagation process in V-groove welded joints exhibits a distinct acceleration characteristic—slow in the initial stage and rapid in the later stage until final fracture.

The crack propagation morphologies and evolution patterns of the specimen models in Figure 18, Figure 19 and Figure 20 are compared, and it is found that the crack propagation modes simulated by the models—including crack initiation sites, propagation paths, and final fracture morphologies—all resemble the observations from low-temperature fatigue tests.

This indicates that integrating the collaborative analysis method of ABAQUS finite element software and FRANC 3D crack propagation simulation software can accurately simulate the fatigue crack initiation, propagation, and fatigue life results during the specimen’s fatigue test. This method is used to conduct exploratory prediction of the fatigue life of weathering steel V-groove welded joint specimens.

**Figure 21:**
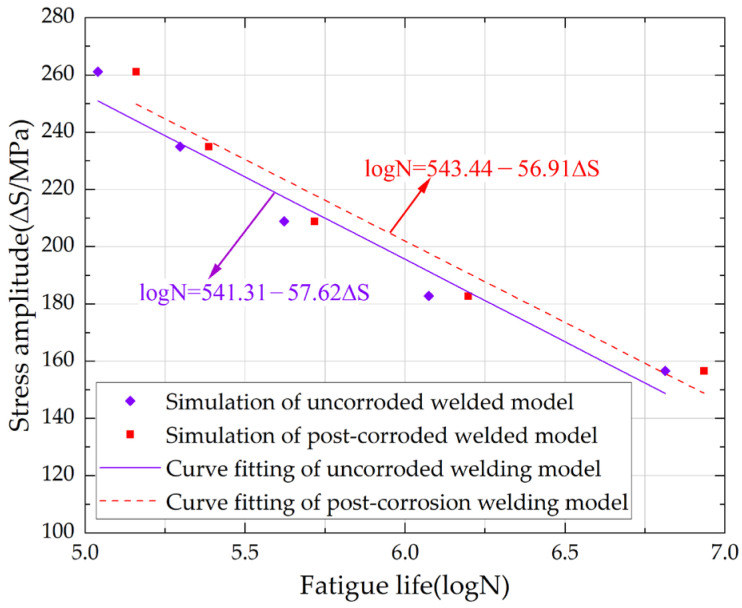
Simulation of S-N curves for low-temperature fatigue in corroded specimens.

To investigate the influence mechanism of environmental factors on the fatigue life of specimens, this study systematically analyzed the S-N curves (Figure 21) under low-temperature corrosive environments based on numerical simulation results. The findings demonstrate that under low-temperature conditions, the fatigue life of specimens exhibits a significant increasing trend with the reduction in stress amplitude (ΔS). When the stress amplitude decreases from 182.70 MPa to 156.60 MPa, the fatigue life of uncorroded specimens increases by 447.70%, and that of specimens corroded for 18 days increases by 445.72%. Comparative analysis revealed that the fitted stress amplitude (ΔS) of corroded specimen models is generally higher than that of uncorroded specimens, indicating that mild corrosion environments can enhance the fatigue resistance of specimens with V-groove welded joints.

From the mathematical characteristics of the S-N curves, the fatigue fitting curves of corroded specimen models exhibit dual features: a decreased slope (reduced m-value) and an increased intercept, further confirming the enhancing effect of mild corrosion on the fatigue performance of V-groove welded joints in low-temperature environments. Mild corrosion forms tiny corrosion pits on the material surface; these pits disperse stress concentration and prevent its localization in a single area. Additionally, the corrosion pits from mild corrosion exhibit a passivation effect, so higher stress is required for crack initiation. Furthermore, mild corrosion removes minor surface defects (such as scratches, inclusions, etc.), which typically act as starting points of fatigue cracks. The analytical results are consistent with the low-temperature fatigue test data obtained through orthogonal experimental methods, jointly validating the improved low-temperature fatigue performance of Q500qENH weathering steel V-groove welded joint specimens under mild corrosion conditions. This study provides an important theoretical basis for fatigue assessment of welded structures in specialized environments.

The low-temperature fatigue test results are compared, and it is found that mild corrosion extends the fatigue life of Q500qENH weathering steel V-groove welded joint specimens. Under low-temperature conditions, specimens subjected to mild corrosion under the same stress level exhibit an approximately 20.00% higher fatigue life than uncorroded specimens.

Conclusions are derived from simulations and confined only to specific conditions: Q500qENH steel V-groove welded joints (16 mm thickness, GMAW-SAW hybrid welding), −40 °C low-temperature environment, and 2.00 × 10^−2^ mol/L NaHSO_3_ corrosive medium. The conclusions should be additionally validated when extended to other steel grades, welding forms, or corrosive environments (e.g., marine atmospheric environments).

## 7. Discussion

An exploratory analysis is conducted on the equivalent conversion relationship between the indoor accelerated corrosion time and the actual plateau exposure corrosion time of weathering steel welded specimens under the same corrosion condition.

The same-group literature [32] reports that sodium bisulfite (NaHSO_3_) corrosion solution is used to conduct cyclic immersion corrosion tests on Q500qENH weathering steel V-groove welded joint specimens, and the corrosion test results are fitted to derive the relationship between corrosion depth and corrosion time (as shown in Equation (6)).

*B* = 0.02D^0.620^ (6)

The definitions in Equation (6) are as follows: B denotes the corrosion depth and D denotes the indoor accelerated corrosion time.

Meanwhile, corrosion kinetics calculation methods are applied, the power function law [35,36] for conversion is integrated, and then the equivalent conversion relationship of corrosion time for Q500qENH weathering steel V-groove welded joint specimens in the Beijing area is derived (as shown in Equation (7)).

logY = −0.464 + 1.409logD(7)

The definitions in Equation (7) are as follows: Y denotes the natural atmospheric exposure corrosion time and D denotes the indoor accelerated corrosion time.

The 18-day indoor accelerated corrosion time of Q500qENH weathering steel V-groove welded joint specimens is substituted into Equation (7), and it is obtained that the 18-day corrosion of the welded specimens corresponds to 20.17 years of outdoor exposure corrosion in the Beijing area. It is noted that this result indicates the damage degree of the welded specimens in the 18-day indoor accelerated corrosion test is equivalent to that of 20.17 years of natural atmospheric exposure in the Beijing area.

Reference is made to the findings of the same-group study [32], which states that the corrosion severity of steel in the Qinghai–Tibet Plateau is lower than that in the Beijing area. Exploratory conclusions are drawn from this study: in the Qinghai–Tibet Plateau, weathering steel specimens require at least 21 years of natural exposure corrosion to achieve a corrosion effect comparable to 18 days of laboratory cyclic immersion corrosion. However, this estimation does not fully account for the unique environmental factors of the plateau (e.g., extreme temperature fluctuations, strong ultraviolet radiation).

Equation (7) is used to equate the damage degree of welded specimens in indoor accelerated corrosion tests to that under outdoor atmospheric exposure corrosion in the Beijing area. The calculation results of this equation are treated as exploratory, and its equivalence scope is focused on the response law of the fatigue performance of welded specimens to corrosion, rather than on the precise equivalence of indoor and outdoor corrosion degrees.

The cyclic immersion corrosion method is adopted to conduct accelerated corrosion tests on weathering steel V-groove welded specimens, as the research cycle for outdoor exposure corrosion of steel is long. The purpose of the test is set to preliminarily explore the variation trend of the long-term outdoor corrosion behavior of weathering steel under the framework of similar conditions within a shorter indoor simulation time, rather than to obtain an exact “indoor–outdoor equivalence” relationship.

Accelerated corrosion tests for weathering steel specimens are designed to simulate the corrosion effects caused by outdoor exposure in specific environments. These tests are used to quickly grasp the basic laws of the actual corrosion behavior of V-groove welded joint components under long-term outdoor exposure and provide a practical application path for the research conclusions of this paper.

Its application scope is limited to Q500qENH weathering steel V-groove welded joints, high-altitude areas where temperature variation exceeds 40 °C and the minimum temperature can drop below −40 °C, and similar high-altitude sulfur dioxide-rich environments.

Fatigue test results based on orthogonal experimental design further reveal that, under natural corrosion conditions, Q500qENH weathering steel V-groove welded joints must undergo a specific corrosion process to achieve optimal fatigue life enhancement in the low-temperature corrosive environment of the plateau. Notably, before reaching the optimal service state, the material is prone to damage accumulation effects.

The results demonstrate that Q500qENH weathering steel subjected to moderate pre-corrosion treatment can more effectively utilize its inherent corrosion-resistant properties, thereby significantly improving fatigue performance. This discovery provides important theoretical foundations and practical guidance for the engineering application of weathering steel in plateau environments.

## 8. Conclusions

Integrate the orthogonal experimental method and conduct high-cycle fatigue tests on V-groove welded joint specimens of corrosion-resistant weathering steel. Determine the optimal influencing factors on specimen fatigue life. Establish a finite element model to comparatively analyze the crack propagation mechanisms in fatigue specimens. Predict the fatigue life of the specimen model. The above lead to the following conclusions:

(1) High-cycle fatigue tests based on orthogonal experimental design revealed that stress level is the most dominant factor affecting the fatigue life of Q500qENH weathering steel V-groove welded joints, followed by corrosion duration and ambient temperature. Analysis of the orthogonal test results shows that when the maximum fatigue stress is 174 MPa, the optimal fatigue test conditions correspond to a corrosion time of 9 d and an ambient temperature of −40 °C, or a corrosion time of 18 d and an ambient temperature of −20 °C; when the maximum fatigue stress is 232 MPa, the optimal fatigue test conditions correspond to a corrosion time of 9 d and an ambient temperature of −20 °C; when the maximum fatigue stress is 290 MPa, the optimal fatigue test conditions correspond to a corrosion time of 18 d and an ambient temperature of 20 °C.
(2) High-cycle fatigue fracture surfaces based on SEM analysis revealed that stress concentration is the primary cause of fatigue crack initiation in V-groove welded joint specimens of Q500qENH weathering steel. The study confirmed that cracks in non-corroded specimens originated from internal defects (slag inclusions/porosity), whereas in corroded specimens, cracks initiated at corrosion pits and tear ridges. The synergistic effect of corrosion pits intensified with prolonged exposure time, promoting damage accumulation in the specimens and accelerating crack initiation.
(3) Finite element software was employed to establish a geometric model with initial cracks for simulation. Integrating fatigue test results, this study investigates Q500qENH weathering steel V-groove welded joint specimens under low-temperature conditions (−40 °C) and equivalent stress levels. The findings reveal that slight corrosion exposure can enhance fatigue life, though damage accumulation tends to occur before reaching optimal service performance. It is acknowledged that there are certain uncertainties in the numerical results of the model in this study, which mainly stem from the variability of material parameters, the simplification of boundary conditions, and the assumptions in the numerical model. The conclusions of this study are limited to applications only to specific materials and structural configurations similar to the specimens used. Further validation of the conclusions is required for other specimen types or different test conditions to avoid potential misinterpretation or misapplication.

## Figures and Tables

**Table 1:** Chemical composition of Q500qENH weathering-resistant steel.

Element/%	Si	C	Mn	S	P	Cr	Ni	Mo	Cu	V	Nb
Q500qENH	0.340	0.060	1.360	0.003	0.013	0.470	0.410	0.100	0.250	0.036	0.025

Note: Each element in the table is expressed in terms of mass fraction, where mass fraction = (mass of the target element/total mass of elements) × 100%.

**Table 2:** Chemical element content of welding electrode for Q500qENH weathering steel welded joint.

Element/%	Si	C	Mn	S	P	Cr	Ni	Mo	Cu
JWER60NHQ	0.360	0.050	1.130	0.002	0.008	0.360	0.550	—	0.340
JWS60NHQ	0.380	0.043	1.610	0.003	0.013	0.330	0.420	—	0.280

Note: Each element in the table is expressed in terms of mass fraction, where mass fraction = (mass of the target element/total mass of elements) × 100%.

**Table 3:** Welding parameters of V-groove welded joint specimens for Q500qENH weathering steel.

Groove Form	Welding Method	Welding Material	Welding Pass	Current/A	Voltage/V	Welding Speed/m·h^−1^
V-groove	GMAW	JWER60NHQ	1	230	28	20
	GMAW	JWER60NHQ	2	280	31	20
	SAW	JWS60NHQ	3–6	610	30	24

**Table 4:** Fatigue test program.

Serial Number	Specimen Number	Weld Toe Reinforcement/mm	Weld Toe Radius/mm	D/d	T/°C	S/MPa
1	PV0-1	≤3	≥3	0	20	174
2	PV0-2			0	−20	232
3	PV0-3			0	−40	290
4	PV1-1			9	20	232
5	PV1-2			9	−20	290
6	PV1-3			9	−40	174
7	PV2-1			18	20	290
8	PV2-2			18	−20	174
9	PV2-3			18	−40	232

Note: The weld toe geometry of the weld falls within the above range, but it was not explicitly controlled as a test parameter.

**Table 5:** Tensile mechanical properties of uncorroded 16 mm thick Q500qENH weathering steel v-groove welded joint specimens.

Sample Specimen Number	E/MPa	*f_y_*/MPa	*f_u_*/MPa	A/%	*f_y_*/*f_u_*	*εu*/%
PV0-1	207,610	580.07	683.48	18.18	0.85	9.59

**Table 6:** Results of orthogonal design experiment.

Sample Specimen Number	D/d	T/°C	S/MPa	Fatigue Life/N
PV0-1	0	20	174	1,319,500
PV0-2	0	−20	232	332,700
PV0-3	0	−40	290	101,400
PV1-1	9	−40	174	2,000,000
PV1-2	9	20	232	2,000,000
PV1-3	9	−20	290	167,700
PV2-1	18	20	290	350,000
PV2-2	18	−20	174	2,000,000
PV2-3	18	−40	232	797,500

Note: PV1-1, PV1-2, and PV2-2 are ‘run-out specimens’; the 2,000,000 fatigue loading cycles represent the test termination cycles of the specimens under the current stress level.

**Table 7:** Range analysis results.

${\bar{y}}_{kn}$	${\bar{y}}_{k1}$	${\bar{y}}_{k2}$	${\bar{y}}_{k3}$	$\mathrm{Max}({\bar{y}}_{kn})$	*R_k_*
D	584,533	1,389,233	1,049,167	1,389,233	804,700
T	966,300	833,467	1,223,167	1,223,167	389,700
S	1,773,167	1,043,400	206,367	1,773,167	1,566,800

**Table 8:** Analysis of variance results.

Influencing Factors	d*f*	SS/×10^11^	MSE/×10^11^	F	Influence Contribution Degree
D	2	9.79	4.90	1.61	II
T	2	2.35	1.18	0.39	III
S	2	36.90	18.40	6.07	I
e	2	6.08	3.04	/	/
Total	8	55.10	/	/	/

Note: The large values of SS and MSE are due to the large numerical range of the fatigue life data (unit: cycles); the squaring of the data values during the calculation process results in the observed large numerical outcomes.

**Table 9:** Test Results of Crack Propagation for Q500qENH Steel at −40 °C.

T/°C	E/MPa	*ρ*/ton·mm^−3^	*μ*	C	m
−40	206,500	7.85 × 10^−9^	0.30	6.97 × 10^−12^	2.75

**Table 10:** Scanning results of corrosion morphology for Q500qENH weathering steel V-groove welded joint specimens.

D/d	Corrosion Depth/mm	Scanned Area	S_a_/μm	S_q_/μm	S_p_/μm	S_v_/μm	S_z_/μm	Sdr/%
9	0.071	WZ	144.22	168.89	376.54	472.34	848.88	25.92
		BMZ	25.89	32.43	81.98	136.16	218.14	21.66
18	0.138	WZ	153.29	181.47	283.75	537.06	820.80	19.67
		BMZ	27.66	34.23	102.50	129.72	232.22	24.71

Note: WZ—Weld Zone; BMZ—Base Metal Zone.

**Table 11:** Simulation of fatigue performance of v-groove welded joint specimens under −40 °C.

Exposure Period/d	Stress Level/MPa	Stress Amplitude ΔS/MPa	Fatigue Life/N	Crack Propagation Depth a/mm	logΔS	logN
0	174	156.60	6,496,247	11.56	2.20	6.81
	203	182.70	1,186,089	11.55	2.26	6.07
	232	208.80	418,499	11.54	2.32	5.62
	261	234.90	197,517	11.57	2.37	5.30
	290	261.00	109,308	11.77	2.42	5.04
18	174	156.60	8,581,337	11.55	2.20	6.93
	203	182.70	1,572,529	11.19	2.26	6.20
	232	208.80	820,672	11.04	2.32	5.72
	261	234.90	243,071	10.73	2.37	5.39
	290	261.00	144,222	10.98	2.42	5.16

**Table 12:** Experimental versus simulated fatigue life comparison.

Specimen Number	Experimental Temperature/°C	Stress Level/MPa	Fatigue Life/N	Simulated Fatigue Life/N	Relative Error
PV0-3	−40	290.00	101,400	109,271	7.76%
PV2-3	−40	232.00	797,500	820,672	2.91%

## Data Availability

The original contributions presented in this study are included in the article. Further inquiries can be directed to the corresponding author.
